# *Plasmodium falciparum* Erythrocytic Stage Parasites Require the Putative Autophagy Protein PfAtg7 for Normal Growth

**DOI:** 10.1371/journal.pone.0067047

**Published:** 2013-06-25

**Authors:** Dawn M. Walker, Najmus Mahfooz, Katherine A. Kemme, Viral C. Patel, Maribeth Spangler, Mark E. Drew

**Affiliations:** 1 Department of Microbial Infection and Immunity, The Ohio State University Wexner Medical Center, Columbus, Ohio, United States of America; 2 Division of Medicinal Chemistry, The Ohio State University College of Pharmacy, Columbus, Ohio, United States of America; Université Pierre et Marie Curie, France

## Abstract

Analysis of the *Plasmodium falciparum* genome reveals a limited number of putative autophagy genes, specifically the four genes involved in ATG8 lipidation, an essential step in formation of autophagosomes. In yeast, Atg8 lipidation requires the E1-type ligase Atg7, an E2-type ligase Atg3, and a cysteine protease Atg4. These four putative *P. falciparum* ATG (PfATG) genes are transcribed during the parasite’s erythrocytic stages. PfAtg7 has relatively low identity and similarity to yeast Atg7 (14.7% and 32.2%, respectively), due primarily to long insertions typical of *P. falciparum*. Excluding the insertions the identity and similarity are higher (38.0% and 70.8%, respectively). This and the fact that key residues are conserved, including the catalytic cysteine and ATP binding domain, we hypothesize that PfAtg7 is the activating enzyme of PfAtg8. To assess the role of PfAtg7 we have generated two transgenic parasite lines. In one, the PfATG7 locus was modified to introduce a C-terminal hemagglutinin tag. Western blotting reveals two distinct protein species, one migrating near the predicted 150 kDa and one at approximately 65 kDa. The second transgenic line introduces an inducible degradation domain into the PfATG7 locus, allowing us to rapidly attenuate PfAtg7 protein levels. Corresponding species are also observed in this parasite line at approximately 200 kDa and 100 kDa. Upon PfATG7 attenuation parasites exhibit a slow growth phenotype indicating the essentiality of this putative enzyme for normal growth.

## Introduction

Autophagy (from Greek *auto*, self+*phagein*, eating) has recently become accepted as an important lysosome-mediated catabolic process in eukaryotes. It serves a diversity of cellular roles such as nutrient acquisition during starvation, protein trafficking (Cvt pathway), anti-apoptosis, and remarkably, the clearance of relatively large macromolecular structures such as protein aggregates, mitochondria, peroxisomes, nuclei, and intracellular pathogens [1]–[11]. Rapid induction of autophagy during starvation provides essential amino acids and energy to synthesize proteins necessary for survival [12]. Misregulation of autophagy has been shown to be involved in human diseases, such as cancer and neurodegenerative diseases [13]–[16].

Many of the proteins involved in autophagy have been identified and characterized in *Saccharomyces cerevisiae*, including approximately 30 autophagy-related (ATG) genes [17]. Despite ATG genes being highly conserved across eukaryotes, there is seemingly only a subset conserved among parasitic protozoa including *Leishmania, Toxoplasma, Trypanosoma and Plasmodium* [18]–[20]. In common to these protozoa are members of the Atg8 lipidation pathway, an essential pathway for formation of autophagosomes, the double membrane vesicles that envelop cargo for delivery to the lysosome. As described in yeast, the small ubiquitin-like modifier Atg8 plays an integral role in this process [21]. Under certain conditions, such as nutrient starvation, autophagy becomes highly upregulated as the E1- and E2- type ligases Atg7 and Atg3 activate cytosolic Atg8 by conjugating it to phosphatidylethanolamine (PE) [22], with a result in an increase in autophagosome formation [23].

*Plasmodium falciparum* is the causative agent of the most deadly form of human malaria and, like many parasites, has multiple developmental stages that are adapted to its two hosts (the human and the anopheline mosquito). Autophagy proteins have been studied in liver stages of the rodent malaria parasite *P. berghei*, where autophagsome-like structures are present and appear to eliminate organelles such as micronemes and mitochondria [24]. To date, there are no published data regarding the essentiality of autophagy proteins in *Plasmodium*, especially during the disease-causing erythrocytic stages of *P. falciparum*.

Our genomic analysis has revealed *P. falciparum* to have a limited repertoire of putative ATG genes present in the genome, the most identifiable being the members of the Atg8 lipidation system. The Atg8 lipidation pathway also appears to be present in other protozoan parasites [18]–[20].

Atg7 is a ubiquitin-related modifier, namely an E1-type activating enzyme. The mechanism of Atg8 lipidation mimics that of protein ubiquitination, which has been well characterized in systems such as yeast and mammals [25]. Briefly, during ubiquitination (or autophagy), a thioester intermediate is formed between the E1 (Atg7) and ubiquitin (Atg8). Ubiquitin (Atg8) is then transferred to the catalytic cysteine residue of the ubiquitin-conjugating enzyme or E2 (Atg3). The final step includes transfer of ubiquitin (Atg8) to its target protein (PE) forming a covalent bond through an isopeptide linkage. This can occur directly by the E2 or through a third ubiquitin-protein ligase or E3 (Atg5-Atg12).

In this study we show that the putative Atg8 lipidation pathway members PfATG3 (PF3D7_0905700.2), PfATG4 (PF3D7_1417300), PfATG7 (PF3D7_1126100) and PfATG8 (PF3D7_1019900) are transcribed in erythrocytic stage parasites. We focus on the putative PfAtg7 because as the activating enzyme of PfAtg8 lipidation, PfAtg7 could have an interesting biological role in the parasite, as well as the potential to be a good drug target, having notable differences from its mammalian counterpart. We confirm PfAtg7 expression by modifying the gene locus to add a C-terminally encoded epitope tag (HA), which reveals the presence of two PfAtg7 species. This suggests a post-translational processing of PfAtg7. We are able to attenuate levels of endogenous PfAtg7 through integration of a C-terminal regulatable fluorescent affinity (RFA) tag that allows for rapid destabilizion of the fusion protein, PfAtg7-RFA. Attenuation of PfAtg7-RFA results in a marked reduction in parasite growth, demonstrating the requirement of PfAtg7 during *P. falciparum’s* erythrocytic cycle for normal growth.

## Materials and Methods

All reagents were purchased from Sigma-Aldrich unless otherwise stated. Human O^−^ erythrocytes, from anonymous donors, were purchased from Interstate Blood Bank (Nashville TN).

### Bioinformatic Analysis

Known yeast Atg protein sequences were obtained from the *Saccharomyces* Genome Database (www.yeastgenome.org). Putative *P. falciparum* proteins were identified by Blastp through PlasmoDB (www.plasmodb.org) using default parameters. Alignments were performed using ClustalW (www.ebi.ac.uk/tools/msa/clustalw2) with default alignment parameters. Percent identity and similarity were calculated by hand using the ClustalW alignment.

### Parasite Culture, Transfection, and Selection

Parasites were maintained and synchronized by standard methods [26], [27]. Culture media included: RPMI (plus L-glutamine, without NaHCO_3_), 0.5% albumax, 0.37 mM hypoxanthine, 27 mM NaHCO_3_, 11 mM glucose, 10 µg/ml gentamicin. Parasites were cultured in O^−^ human erythrocytes at 2% hematocrit under 5% CO_2_, 5% O_2_, and 90% N_2_, 37°C. For transfections, 400 µl of 50% hematocrit RBCs infected with ring stage parasites was transfected with 100 µg of purified plasmid DNA by electroporation, as previously described [28]. Atg7-HA was transfected into clone 3D7 parasites and PfAtg7-RFA was transfected into the PM1 cell line, which expresses human DHFR conferring resistance to TMP [29], [30]. Transfected parasites were selected with WR99210 or blasticidin (Atg7-HA and Atg7-RFA, respectively) with Atg7-RFA parasites maintained continuously in 5 µM TMP. Following two rounds of on-off drug cycling to enrich for integrants, clonal lines were obtained by limited dilution into 96-well plates. Southern blotting confirms proper integration (Fig. S2 in File S1).

### Amplification of mRNA

RNA was purified using Trizol (Invitrogen) and cDNA was synthesized using Superscript II reverse transcriptase (Invitrogen) by heating 10 µg total RNA with oligo-dT and dNTPs at 65°C, 5 min followed by addition of RT buffer and incubated at 42°C, 2 h. DNAse-free RNase was added and incubated for 30 min, 37°C. The reaction was inactivated by heating at 65°C for 10 min. Standard PCR conditions were used with annealing performed at 50°C. Primers were (forward and reverse, respectively):

PfATG4: 5′-AGATCTTCGGACTAGTCAGAGGAA-3′ and 5′-ATCGATAACTCCATGGCGCTGGTT-3′.

PfATG3: 5′-CCATGGTCAGAGGAAATTTTTGAGGA-3′ and 5′-CCTAGGTCATAATCCTGCATTGCTAT-3′.

PfATG7: 5′-CCATGGTTTTTGCTCCCCATATAGAC-3′ and 5′-CCTAGGTCATTCCAATATTATAACAT-3′.

PfATG8 5′-GGATCCCCATCGCTTAAAGACGAA-3′ and 5′-GTCGACTTATCCTAGACAACTCTCAC-3′.

Genomic DNA was purified from parasites using QIAamp DNA blood mini kit (Qiagen) and used as a control. Predicted sizes of amplified products: PfATG3: 282 bp for cDNA, 643 bp for gDNA; PfATG4: 740 bp for cDNA, 1030 bp for gDNA; PfATG7: 333 bp for cDNA, 512 bp for gDNA; PfATG8: 375 bp for both cDNA and gDNA.

### Plasmid Construction

PfAtg7-HA: A targeting fragment comprising the 3′ end of the PfATG7 gene was amplified using the primers CTCGAGGGTGATAATGTGTTATGTGA and CCTAGGTTCCAATATTATAACATCATT with Platinum Taq (Invitrogen) and cloned using the TOPO-TA for Sequencing kit (Invitrogen). DNA sequencing was performed (Nucleic Acid Sequencing facility at the Ohio State University) to confirm proper sequence, followed by release of the insert through digestion with XhoI and AvrII and ligated into pPM2GT-HA [31]. For the PfAtg7-RFA construct, the PfATG7 targeting fragment (above) was cloned into a modified version of pGDB in the same manner [30].

### Growth Analysis

Growth experiments of PfAtg7-RFA clones were initiated at 1% parasitemia, 1 or 2% hematocrit in appropriate culture media with or without TMP. Parasitemia was monitored daily by flow cytometry. All growth experiments were carried out multiple times in triplicate.

### Flow Cytometry

Flow cytometry was used to assess the percentage of infected RBCs (parasitemia) by staining live cells with 1.5 µg/ml acridine orange in PBS, 5 min, RT. The increase in fluorescence of infected RBCs was detected using a FACSCanto II flow cytometer (BD Biosystems) and parasitemia enumerated by gating of the high fluorescence cell population. 30,000 cells were analyzed for each sampling.

### Parasite Purification, Western Blotting, and Antibodies

All incubations were performed at RT unless otherwise noted. Parasites were harvested for western blot analysis using saponin release (0.025% in PBS). To avoid proteolysis, all harvests were performed rapidly on ice and parasites were washed with ice-cold PBS with Complete protease inhibitor (Roche USA). Parasites were mixed with loading buffer (250 mM Tris-HCl, pH 6.8, 6% SDS, 20% β-mercaptoethanol, 0.04% bromophenol blue, 40% glycerol), boiled for 5 min, and centrifuged at 14,000 RPM for 5 min to remove insoluble material. Lysates representing 1×10^7^ parasites were resolved on 4–20% Criterion SDS-PAGE gels (Bio-Rad) and transferred to methanol-activated polyvinylidene fluoride membrane (Millipore) using transfer buffer (20 mM Tris-HCl, 192 mM glycine, 20% methanol) via the TransBlot Semi-Dry Transfer system (Bio-Rad). The membrane was blocked with 5% nonfat milk powder in 1× TBST (20 mM Tris-HCl, 150 mM NaCl, 0.1% Tween20) for 30 min. Primary antibody dilution was made in 5% nonfat milk in TBST and incubated with the membrane overnight at 4°C. Secondary antibodies were diluted in TBST and membrane was incubated with secondary for 1 hour. All washes were 3×10 min in TBST. Immunoblots were developed using the Supersignal West Dura chemiluminescent substrate (Thermo Scientific). An independent transgenic *P. falciparum* UCH-HA expressing an unrelated HA tagged protein was used as a control for HA western blotting. Antibodies and dilutions were: affinity purified anti-HA, rabbit, 1∶5000 (Rockland); anti-rabbit IgG horseradish peroxidase-linked whole antibody, 1∶10000 (GE Healthcare); anti-Plasmodium plasmepsin V, 1∶1000 (Goldberg Lab, Washington University, St. Louis MO), α-mouse IgG horseradish peroxidase-linked whole antibody, 1∶5000 (GE Healthcare).

### Densitometry

Percent attenuation of PfAtg7 was assessed by densitometry and normalized to PMV to account for loading. Pixel intensities of scanned images were determined using ImageJ software (NIH).

## Results

### Atg8 Lipidation Genes are Expressed during P. falciparum Erythrocytic Stages

Over 30 autophagy-like (ATG) genes and other molecular components of autophagy have been discovered, largely in *Saccharomyces cerevisiae* [17]. To identify putative autophagy machinery in *P. falciparum*, Atg protein sequences from *S. cerevisiae* were used in homology searches through the PlasmoDB database. The results of this search were generally consistent with other bioinformatic studies [18]–[20]; however, a number of differences were obtained in our analysis which yielded a limited repertoire of putative autophagy genes (Fig. S1C and Table S1 in File S1). This limited number of ATG genes included the four genes involved in Atg8 lipidation: ATG8, ATG4, ATG7, ATG3. This ubiquitin-like conjugation pathway is involved in autophagosome formation [22], [32]. The comparison between yeast and *P. falciparum* Atg8 yields the highest similarity of the four proteins: Atg8, Atg4, Atg3, Atg7. Both PfAtg4 and PfAtg7, when compared to yeast, are predicted to encode long insertions, which are commonly found in *P. falciparum* proteins [33]–[36].

We were unable to detect a similar Atg5 in *P. falciparum* despite the presence of a low similarity, putative Atg12. In yeast, Atg5-Atg12 conjugation is essential for autophagosome formation but has been shown dispensable *in vitro* [37]. The presence of a PfATG12 but not other members of this arm of the autophagy pathway suggests they were lost evolutionarily or other gene products have assumed their function. The related apicomplexan parasite *Toxoplasma gondii* also appears to lack the homologs to the Atg5 and Atg12 [38], [39].

**Figure 1:**
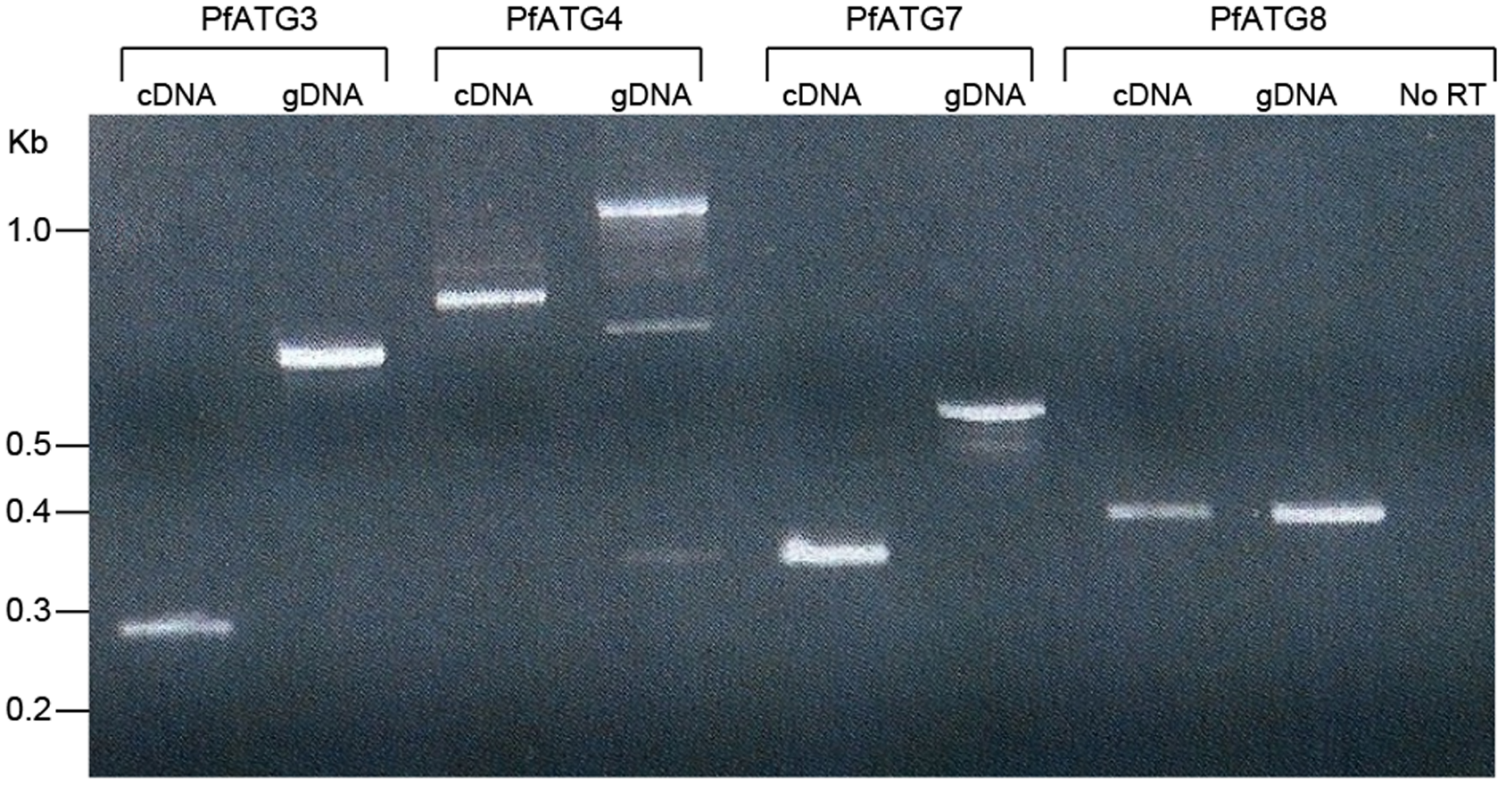
PfAtgs 3, 4, 7, 8 are expressed in*P. falciparum erythrocytic stage parasites.* Genomic DNA (gDNA) and RNA was extracted from asynchronous erythrocytic stage parasites, the latter used for cDNA production. Primers flanking introns were chosen, except for PfATG8, which has no predicted introns. No RT control shows lack of gDNA contamination of RNA preparation.

Published microarray data indicates all four putative autophagy proteins to be expressed during erythrocytic stages of *P. falciparum* infection [40], [41]. To confirm that these genes are transcribed, we purified total RNA from asynchronous 3D7 parasites and synthesized cDNA. Amplification of the cDNA across a predicted intron confirms transcription of PfATG3, PfATG4 and PfATG7 (Fig. 1). PfATG8 has no introns so primers amplify the entire gene.

### Bioinformatic Analysis Indicates Conservation of PfAtg7

**Figure 2:**
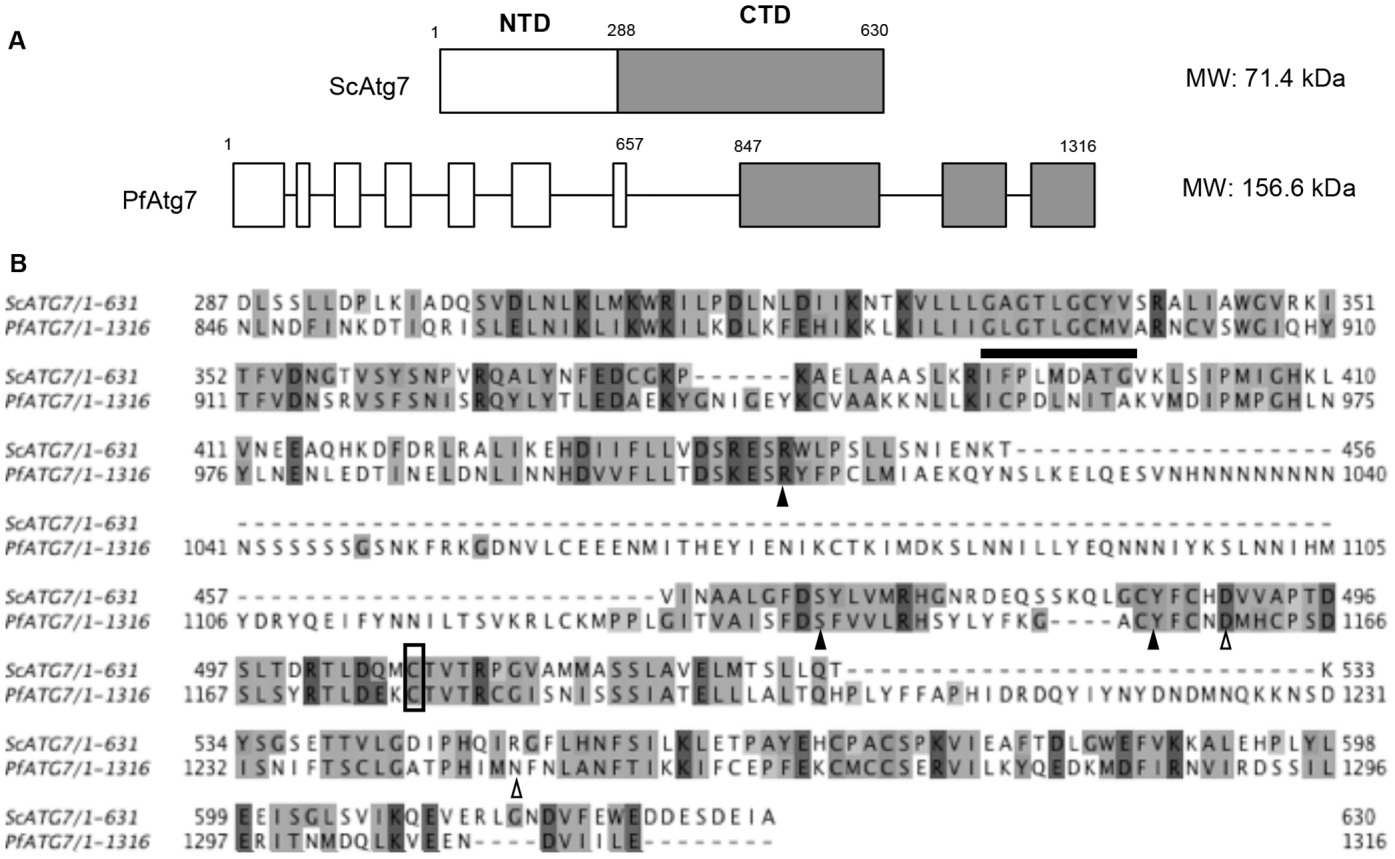
PfAtg7 has an unusual primary structure. (A) PfAtg7 contains long insertions within the C-terminal and N-terminal domains. A schematic of PfATG7 and ScATG7 domains illustrates the presence of insertions in PfAtg7 as compared to the well-described C-terminal and N-terminal domains of ScAtg7 [42]. (B) Alignment of ScAtg7 and PfAtg7 C-terminal regions reveals conservation of key motifs. Alignment between yeast (Sc) and *P. falciparum* (Pf) ATG7 C-terminal domain illustrate similarity, with conservation of the ATP binding domain (black bar), the catalytic cysteine (box), amino acids required for hydrogen bonding (black arrowheads), and salt bridges (open arrowheads) between ATG7 and ATG8 (for complete alignment see Fig. S1C in File S1).

PfAtg7 appears to be significantly larger than E1-type ligases in other systems (Fig. 2A). The enzyme in *Plasmodium* is predicted to be 156.6 kDa whereas in yeast ScAtg7 is 71.4 kDa. ScAtg7 has an N-terminal domain comprising the first 288 amino acids, a 6 amino acid linker region, and a 336 amino acid C-terminal domain containing the catalytic core and dimerization domain [42]. Taking *P. falciparum’s* apparent insertions out of consideration, PfAtg7 has 30.8% identity and 68.1% similarity to ScAtg7. The C-terminal domain itself, again taking the two long insertions out of consideration, has 38.0% identity and 70.8% similarity (Fig. 2B). The C-terminal 123 amino acids, required for dimerization, is well conserved, although there is a unique 29 amino acid insertion present in *P. falciparum* that is absent in yeast. Also conserved is the catalytic cysteine, in yeast at position 507 (1177 in *P. falciparum*) within the essential C-terminus. The ATP binding domain is conserved with a motif of GxGxxGCx at position 890–897. In yeast the residues Y486, R443, and S466 are indicated to be necessary for the formation of hydrogen bonds between Atg7 and Atg8 [42]. The corresponding residues Y1156, R1008, and S1140 are conserved in PfAtg7. In yeast, residues significant for salt bridge formation between Atg7 and Atg8 include: D490 and R550. Only the aspartic acid at 1160 is conserved in *P. falciparum*.

### PfAtg7 is Detected as Two Protein Species

To begin our studies of PfAtg7 in the parasite, a transgenic line was generated in which we modified the gene locus, via homologous recombination, to encode for a C-terminal hemagglutinin (HA) epitope tag on the protein. For this, a targeting plasmid was constructed which included approximately 1 kb of the 3′ end of the gene followed in-frame by the coding sequence to add the HA tag. Clonal integrant lines C1 and F2 were isolated by limiting dilution and proper integration into the PfATG7 locus was confirmed by Southern blotting (Fig. S2 in File S1).

**Figure 3:**
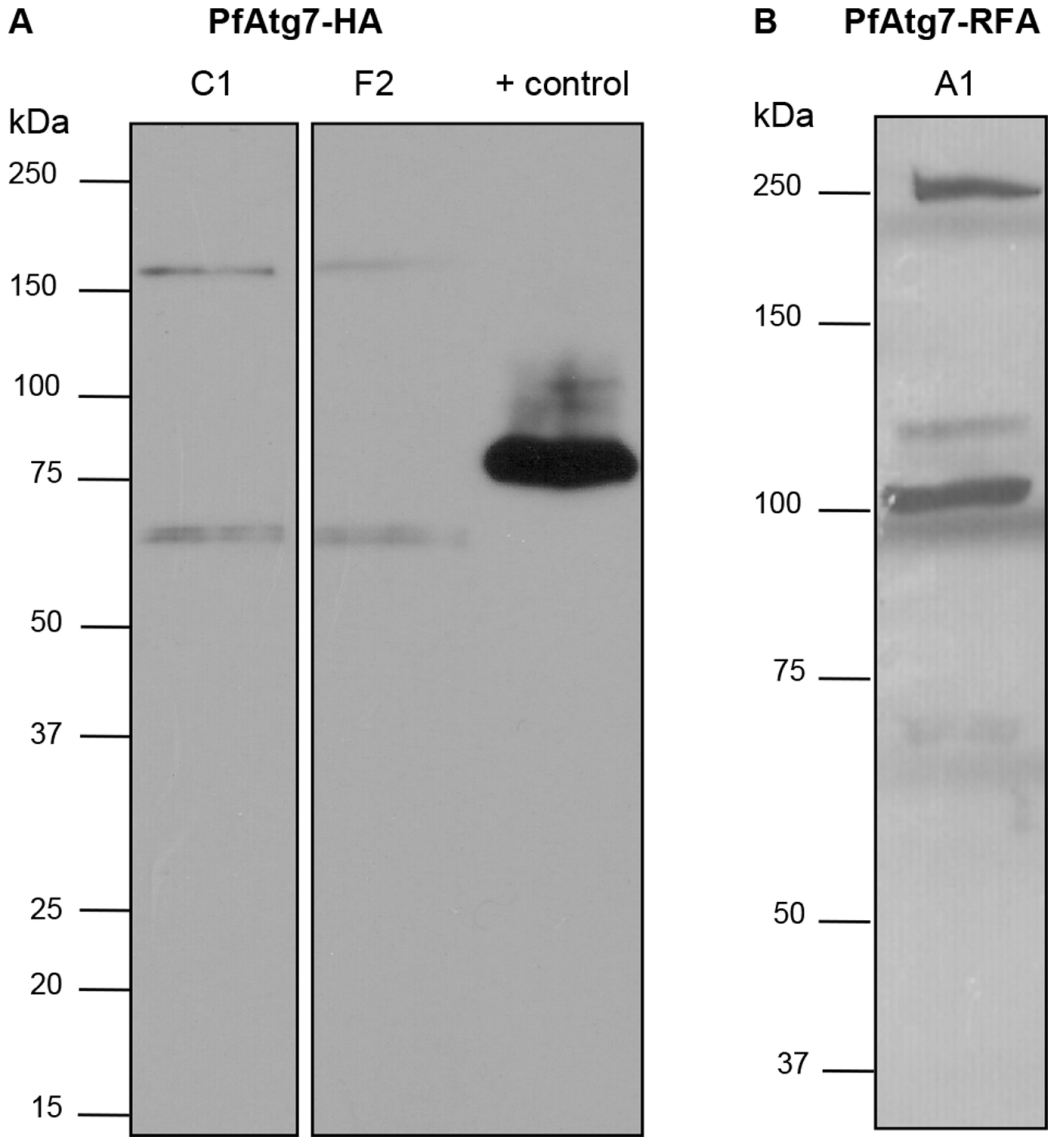
Western blotting confirms expression of PfAtg7 fusion proteins. (A) PfATG7-HA transgenic parasites confirm protein expression. Western blot of two independent clones detects PfAtg7-HA at ∼150 kDa and ∼65 kDa. Lane 1: PfAtg7-HA clone C1; Lane 2: PfAtg7-HA clone F2; Lane 3: UCH-HA positive control (see Materials and Methods). (B) PfAtg7-RFA transgenic parasites confirm expression of the two PfAtg7 species. Western blot detection of PfATG7-RFA fusion protein incubated in the presence of TMP reveals PfATG7 at ∼200 kDa and ∼100 kDa. RFA tag is 47 kDa.

Western blotting of total parasite lysate against the small 9 amino acid HA epitope of the resultant fusion protein, under control of the native promotor, confirms expression of the protein during the erythrocytic stages of the parasite’s life cycle (Fig. 3A). Unexpectedly, we consistently detect two species, one near the predicted size of the full-length protein at ∼150 kDa and one at ∼65 kDa. Despite all efforts to minimize proteolysis (see Material and Methods), these two species are consistently and repeatedly detected. We have yet to confirm which species have enzymatic activity. It is notable that these species might be artifactual as a result of the HA epitope tag.

### Attenuation of PfATG7 Results in Slow Parasite Growth

**Figure 4:**
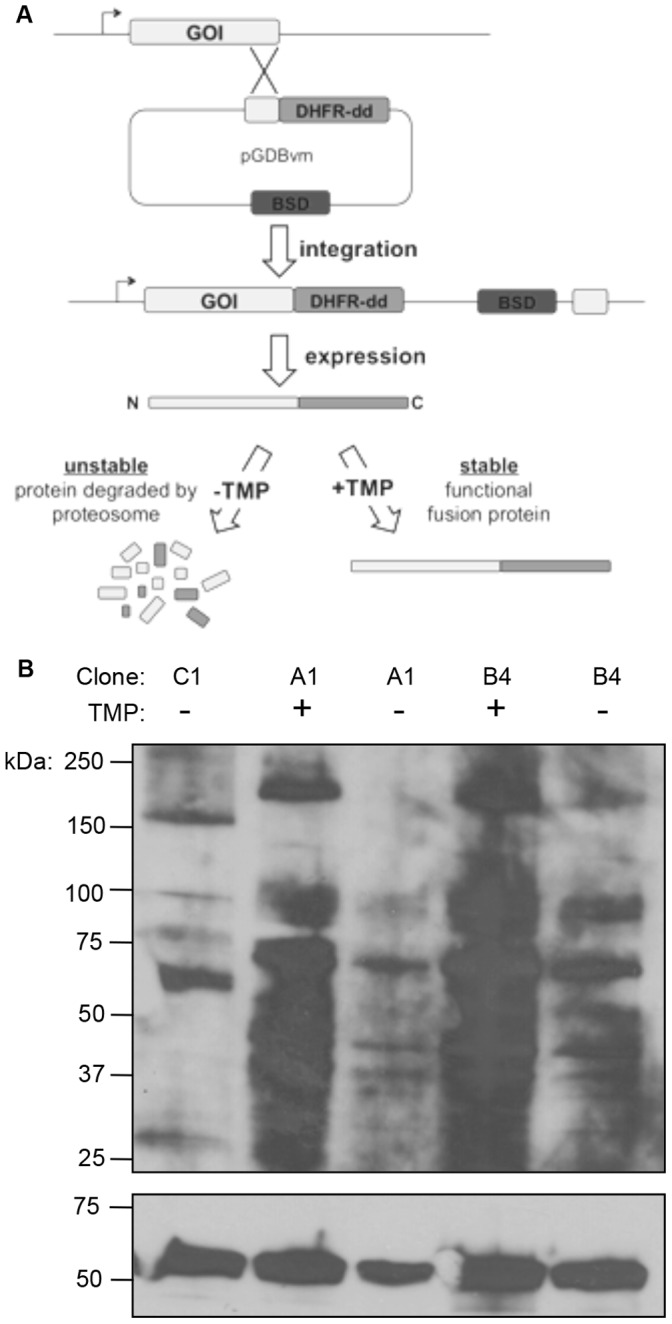
Use of regulatable fluorescent affinity (RFA) tag in*P. falciparum* to attenuate PfATG7. (A) RFA tagging scheme. A fragment representing ∼1 kb of the 3′ end of the gene of interest (GOI) is amplified by PCR and cloned into an integration plasmid designed to undergo homologous recombination with the parasite’s endogenous GOI, resulting in the addition of extra coding sequence at the 3′ end of the gene. This construct adds a C-terminal RFA tag which results in the GOI fused to an attenuatable destabilization domain, which is regulated by trimethoprim (TMP) concentration, in addition to a GFP tag and an HA epitope tag. (B) Western blot of PfATG7-RFA transgenic parasites confirm protein attenuation. Following 48 hours post TMP washout during the growth assay (Fig. 5D), PfAtg7 protein levels were attenuated. The ER-resident Plasmepsin V was used as a control to normalize for loading. Densitometry indicates that the ∼200 kDa band in clone A1 was attenuated by 67.6% (±4.0%) and the ∼100 kDa band was attenuated 17.0% (±2.4%). In clone B4 the ∼200 kDa band was attenuated by 36.1% (±8.5%) and the ∼100 kDa band was attenuated 8.0% (±1.9%).

In a strategy similar to that described above, we tagged PfAtg7 with a regulatable fluorescent affinity tag (RFA), an approach that has been successful for regulated protein attenuation in *P. falciparum* [30], [43]. This construct adds the RFA tag to the C-terminus, which results in the Atg7 protein fused to an attenuable destabilization domain (in addition to GFP and HA), which is stabilized by the presence of the folate analog trimethoprim (TMP) in the growth medium. In the absence of TMP the fusion protein becomes misfolded and rapidly degraded via the proteasome (Fig. 4A). The PfATG7-RFA construct was successfully integrated into the genome of the parental PM1 cell line which expresses human dihydrofolate reductase (DHFR) conferring resistance to TMP [29], [30]. Clonal cell lines A1 and B4 were isolated for further analysis and proper integration was confirmed by southern blot (Fig. S2 in File S1).

Western blotting against the HA epitope present within the RFA tag, reveals two species at ∼200 kDa and ∼100 kDa. Given the addition of the larger 47 kDa tag this corresponds to the two forms observed in the Atg7-HA cell lines (Figs. 3B, 4A,B), which decreases the suspicion that these are merely artifacts. Both protein species were rapidly and sustainably lost upon removal of TMP, with attenuation observed as quickly as 4 hours post TMP removal (data not shown) and this loss was shown to be sustained for 48 h (Fig. 4B). It is likely the loss of PfAtg7 was sustained throughout the growth experiment.

**Figure 5:**
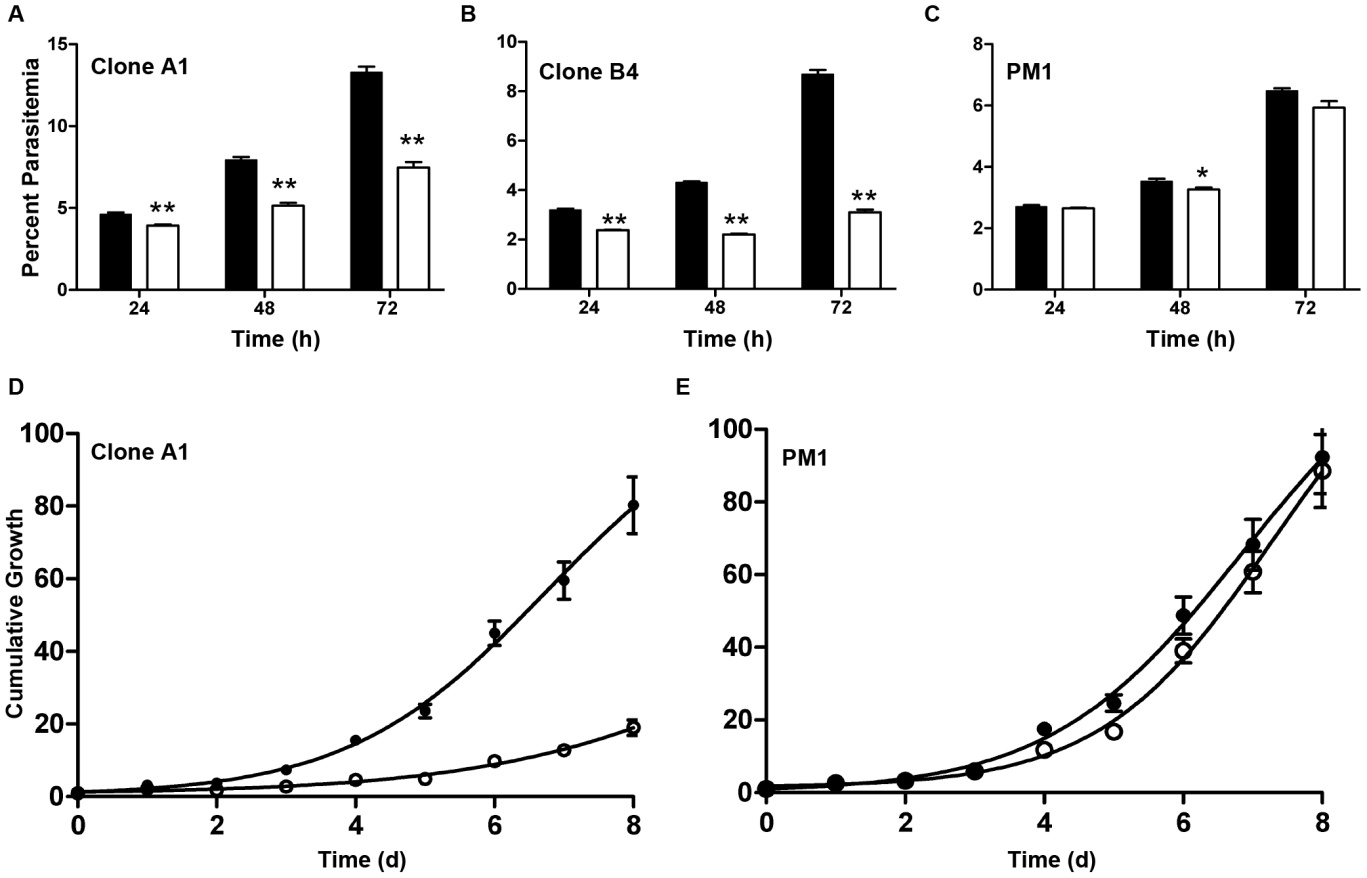
PfAtg7 is essential for normal parasite growth. Short-term growth experiment reveals slow growth phenotype upon TMP removal. PfAtg7-RFA clone A1 (A) B4 (B) and parental PM1 (C) were washed free of TMP and parasitemia was monitored by flow cytometry every 24 h for 3 days. Parasitemia in the absence of TMP (white bars/circles) for clones A1 and B4 was significantly reduced compared to growth in the presence of TMP (black bars/circles; **p<0.001, student’s T test). Parental line (PM1) shows significant growth difference at 48 h (*p<0.05, student’s T test) but no significant difference in the presence and absence of TMP at the 24 h or 72 h timepoints. (D) PfATG7 attenuation results in a sustained slow growth phenotype. Two independent PfATG7-RFA transgenic clones, A1 and B4 (not shown), were monitored every 24 h by staining with acridine orange and enumeration of parasitemia by flow cytometry for up to 8 days. Dilutions were performed on parasite cultures every three days to maintain the parasites at optimal parasitemia and avoid parasite death. Overall dilutions were factored in, resulting in “cumulative growth.” (E) PM1 parental parasites exhibit no significant change in growth over the 8 day period.

Loss of PfAtg7 results in a slow growth phenotype, as seen by monitoring parasitemia over time (Fig. 5). Clonal cell lines A1 and B4 were washed free of TMP and parasitemia was measured by flow cytometry for short periods of time without splitting the parasites (Figs. 4A,B, and C) or for a longer growth analysis requiring dilutions every three days (Figs. 4D and E). For these experiments, the overall dilution was taken into account, resulting in “cumulative growth” (Figs. 4D and E).

## Discussion

The study of autophagy in eukaryotes has revealed a functional complexity well beyond the initial discoveries of its roles in lysosomal protein trafficking and as a defense against nutrient starvation. It is now appreciated as a tightly regulated and selective catabolic machine capable of delivering a remarkable variety of cargo to the lysosome. In a homeostatic role, long-lived and damaged proteins, as well as whole organelles are degraded via incorporation into autophagosomes. Selective delivery of lysosomal resident proteins and other cargo can also occur through what is known as the cytoplasm-to-vacuole (Cvt) targeting pathway [3]. Cellular defense, to both internal and external insult, also appears to be an important role for autophagy. Potentially harmful protein aggregates are enveloped and catabolized, essential nutrient is scavenged from the cytoplasm during periods of metabolic stress, and intracellular pathogens are killed via autophagic mechanisms [6], [11], [12]. Defects in, inhibition of, or overstimulation of autophagy can be either deleterious or protective in a variety of the pathologies caused by cancers, neurodegenerative disorders, heart disease, autoimmune conditions, and intracellular pathogens [11], [14], [44]–[46].

The function of autophagy in protozoan parasites is only beginning to be understood. A response to nutrient deprivation and a possible role in endocytosis has been observed for ATG8 in *L. major*, regulation of cellular differentiation in *T. brucei,* and a role in mitochondrial maintenance and tachyzoite development have been described in *T. gondii* [38], [39], [47], [48]. In *T. gondii*, TgAtg8 localizes to autophagosomes upon starvation and down regulation of TgAtg3 impairs normal development of tachyzoites [39]. Mitochondrial defects are also apparent in *Toxoplasma* upon PfAtg3 attenuation, suggesting a mitophagy-related function [38]. Recent localization of lipidated Atg8 to the apicoplast of *P. falciparum* [49] suggests to us a role in the turnover, maturation, or segregation of this essential organelle.

In contrast to autophagy in more complex eukaryotes, protozoan parasites appear to encode a comparatively small repertoire of autophagy genes. Through bioinformatic analysis, the only autophagy genes common across protozoa appear to be those encoding Atg8 and its lipidation pathway. *Leishmania major* contains four different ATG8 genes, with apparent roles in starvation induced autophagy as well as endocytosis [47]. *L. major* also encodes members of the Atg5-Atg12 conjugation system. Although dispensable *in vitro,* it has been reported that Atg5-Atg12 can act as an E3-type ligase that aids in Atg8 conjugation to PE [37], [50]. Apicomplexan parasites such as *T. gondii* and *P. falciparum,* do not appear to have homologs of the Atg5-Atg12 pathway (a low similarity gene in *P*. *falciparum*, gene ID PF3D7_147000 could be an ATG12) in their genomes but do have putative Atg8 lipidation proteins [18]–[20].

Our expression studies detected mRNA for ATGs 3, 4, 7, and 8 during the erythrocytic stages of *P. falciparum* (Fig. 1), which agrees with published microarray analysis [40], [41]. Each transcript was easily detected and splicing appears to be as predicted by PlasmoDB (with exception of ATG8 which contains no predicted introns). PfAtg7, though putative, has significant domains and key amino acid residues conserved to suggest its functional enzymatic role as an E1-type ligase in PfAtg8 lipidation. Taking out the long insertions, PfAtg7 has 38.0% identity and 70.8% similarity to ScAtg7, with conservation of the catalytic cysteine and the ATP binding domain.

To begin our functional studies on Atg7 in *P. falciparum*, we took a molecular genetic strategy. Western blotting of our Atg7-HA cell lines revealed the presence of two protein species (Fig. 3A). Despite careful attention to minimize the chances of non-specific proteolysis (see Materials and Methods), the smaller species persisted in all experiments. We also observed the same pattern in our ATG7-RFA cell lines (Fig. 3B) consistent with the addition of the larger 47 kDa RFA tag. We speculate the smaller 65 kDa species represents a specific processing event of Atg7, and would be predicted to retain catalytic activity. Pulse-chase experiments and *in vitro* activity assays to examine processing and catalytic activity of these two species are in progress.

Attenuation experiments in our cell lines displayed rapid reduction of PfAtg7 and a significant reduction in parasite growth rate, demonstrating the necessity of PfAtg7 for parasite growth. This reduction was detected in clonal lines as early as 24 h and persisted for as long as 8 days (Fig. 5). This finding is also supported by recent work from the Adams lab who report a ∼3.7-fold reduction in growth of a clonal line of 3D7 *P. falciparum* (the same strain used as a background in our studies) into which random integration of a transposable element occurred 705 bp upstream of the start codon for the ATG7 gene [51]. We confirmed by semi-quanatative RT-PCR that insertion of the transposon results in reduced ATG7 transcription (Fig. S3 in File S1), thus phenocopying the impaired growth as seen in our ATG7-RFA clones upon Atg7 attenuation. It is notable that the parasites did not die upon PfAtg7 attenuation. We speculate this to be due to insufficient attenuation of PfAtg7 or possibly the parasite is able to survive in its absence.

Recently a specific inhibitor of the human E1-type ligase NAE (NEDD8 activating enzyme) has been developed that takes advantage of E1’s enzymatic mechanism [52], [53]. This inhibitor, MLN4924 (Millenium Pharmaceuticals), has led toward development of an anti-cancer drug targeting the NEDD8 pathway that is currently in clinical trials. MLN4924 is an adenosine sulfamate analog that binds to the ATP binding domain, forming an adduct with NEDD8, a small ubiquitin-like protein (as is Atg8). The adduct MLN4924-NEDD8 mimics the NEDD8-adenylate intermediate and binds at the adenylation site of NAE forming a tight binary complex that inhibits NAE from binding ATP or NEDD8, inhibiting conjugation of NEDD8 to its target protein. Thus, it is not unreasonable to suggest that PfAtg7, also an E1-type ligase, may in similar fashion be a druggable target to treat malaria.

The role of autophagy in *Plasmodium* has yet to be elucidated. Recent studies in *Apicomplexa* point to both the apicoplast and mitochondrion as sites of localization of Atg8, leading to our hypothesis of Atg8 involvement the maintenance (e.g., turnover, expansion, segregation) of these essential organelles. The findings of our study support these hypotheses by validation of the putative autophagy protein PfAtg7 and, therefore the PfAtg8 lipidation pathway, as essential for normal growth of the parasite. Our continuing research has the goal of addressing this hypothesis. The validation of PfAtg7 as the activating enzyme of PfAtg8 lipidation and functional analysis of the pathway in parasites are ongoing. In other systems, Atg7 is also involved in the Atg5-Atg12 conjugation pathway; however, our bioinformatic analysis has confirmed the absence of a PfAtg5 in the genome. However, this does not discount that PfAtg7 may be involved in other roles and it is our goal to fully elucidate the role of PfAtg7 in erythrocytic stages of *P. falciparum’s* life cycle.

Whether such an apparent low complexity pathway in these anciently diverging eukaryotes represents the evolutionary origins of autophagy or a divergence from other eukaryotes is still not known, but it is likely that a detailed understanding of “simple autophagy” in *Plasmodium* has potential to enrich our understanding of its role in this medically important parasite as well as benefit our understanding of more complex autophagic systems as in humans.

## Supporting Information

Files S1 Figure S1, Alignments show similarity between yeast and *P. falciparum* Atg proteins. Atg8 (A), Atg4 (B), Atg7 (C) and Atg3 (D) alignments between *P. falciparum* and *S. cerevisiae* performed through ClustalW. PfAtg protein sequences identified by a PlasmoDB blastp using ScAtg protein sequences as described in M&M in the main manuscript. Figure S2, Southern blot confirms integration into correct locus. DNA was purified from transgenic parasites: PfATG7-RFA (clone A1) and PfATG7-HA (clone C1). Southern blot analyzed 1.5 ug of DNA and single cross-over integrations were screened by AvrII/BclI digestion and probed using PfAtg7 ORF 3′ end (1 kb). The expected fragment for 3D7 and PM1 parasites is 4039 bp and the expected size fragments for successful integration of PfATG7-HA are 7653 bp and 3635 bp and for PfATG7-RFA parasites are 7528 bp and 3635 bp. Lack of wildtype locus in PfAtg7-HA and PfAtg-RFA clones indicates successful integration. High intensity band in the PfAtg7-RFA clone possibly represents concatamerization of the plasmid prior to integration into the genome. Figure S3, Semi-quantitative PCR confirms lower expression levels of PfAtg7 in PB-57 parasites. Standard PCR conditions were used to establish expression levels of PfAtg7 (A) and the ribosomal protein upstream of PfAtg7 (B) across a dilution series of cDNA (nanogram amounts) for 3D7 and PB-57 parasites, with quantification also shown (C,D). The ribosomal protein is used as a control as the transposable element was inserted between it and PfAtg7. Predicted sizes of amplified products: PfATG7: 566 bp for cDNA, 745 bp for gDNA; ribosomal protein: 464 bp for cDNA and 700 bp for gDNA. Table S1, Results of bioinformatic analysis of ATG genes in *P. falciparum.* See M&M in main manuscript for search parameters.
(PDF)
