# Supplementary material for: Plasmodium falciparum Erythrocytic Stage Parasites Require the Putative Autophagy Protein PfAtg7 for Normal Growth
Source: PLoS One. 2013 Jun 25;8(6):e67047. doi: 10.1371/journal.pone.0067047 (PMC3692556; doi:10.1371/journal.pone.0067047)
Supplement: Files S1 — Figure S1, Alignments show similarity between yeast and P. falciparum Atg proteins. Atg8 (A), Atg4 (B), Atg7 (C) and Atg3 (D) alignments between P. falciparum and S. cerevisiae performed through ClustalW. PfAtg protein sequences identified by a PlasmoDB blastp using ScAtg protein sequences as described in M&M in the main manuscript. Figure S2, Southern blot confirms integration into correct locus. DNA was purified from transgenic parasites: PfATG7-RFA (clone A1) and PfATG7-HA (clone C1). Southern blot analyzed 1.5 ug of DNA and single cross-over integrations were screened by AvrII/BclI digestion and probed using PfAtg7 ORF 3′ end (1 kb). The expected fragment for 3D7 and PM1 parasites is 4039 bp and the expected size fragments for successful integration of PfATG7-HA are 7653 bp and 3635 bp and for PfATG7-RFA parasites are 7528 bp and 3635 bp. Lack of wildtype locus in PfAtg7-HA and PfAtg-RFA clones indicates successful integration. High intensity band in the PfAtg7-RFA clone possibly represents concatamerization of the plasmid prior to integration into the genome. Figure S3, Semi-quantitative PCR confirms lower expression levels of PfAtg7 in PB-57 parasites. Standard PCR conditions were used to establish expression levels of PfAtg7 (A) and the ribosomal protein upstream of PfAtg7 (B) across a dilution series of cDNA (nanogram amounts) for 3D7 and PB-57 parasites, with quantification also shown (C,D). The ribosomal protein is used as a control as the transposable element was inserted between it and PfAtg7. Predicted sizes of amplified products: PfATG7: 566 bp for cDNA, 745 bp for gDNA; ribosomal protein: 464 bp for cDNA and 700 bp for gDNA. Table S1, Results of bioinformatic analysis of ATG genes in P. falciparum. See M&M in main manuscript for search parameters. (PDF) [file pone.0067047.s001.pdf]

**Supplementary Data**

Supplementary Material and Methods:

*Southern Blot:*

Genomic DNA for southern blots was isolated by QIAamp DNA Blood mini kit (Qiagen). For Southern blots, 1.5 µg of DNA was digested and analyzed as previously described[1]. The single cross-over integrations were screened by AvrII/BclI digestion and probed using PfAtg7 ORF 3' end (1kb). Probe was synthesized by PCR using the AlkPhos direct Labeling system (GE Amersham) with the following primers and developed using CDPStar developing kit (Roche Diagnostics):
For: 5'-CTCGAGGGTGATAATGTGTTATGTGA-3'
Rev: 5'-CCTAGGTTCCAATATTATAACATCATT -3'

*Semi-quantitative PCR:*

*Amplification of mRNA -* RNA was purified using Trizol (Invitrogen) and purified using RNAeasy kit (Qiagen). cDNA was synthesized using Superscript II reverse transcriptase (Invitrogen) by heating 10 µg total RNA with oligo-dT and dNTPs at $65^{o}$ C, 5 min followed by addition of RT buffer and incubated at $42^{o}$ C, 2 h. DNAse-free RNase was added and incubated for 30 min, $37^{o}$ C. The reaction was inactivated by heating at $65^{o}$ C for 10 min.

*Semi-quantitative PCR-* Standard PCR conditions were used on a dilution series of the cDNA with annealing performed at $47^{o}$ C. Primers were (forward and reverse, respectively):

PfATG7 (PF3D7_1126100): 5'-CCTCCTTTAGGTATTACGGTAG -3' and 5'-CCTAGGTCATTCCAATATTATAACAT-3'.
Ribosomal protein (PF3D7_1126200): 5'-GTCACTACAAGTAATAGATAATAATG -3' and 5'-CTCCTTTCTTTTTGGCAACAC -3'.
Predicted sizes of amplified products: PfATG7: 566bp for cDNA, 745bp for gDNA; ribosomal protein: 464bp for cDNA and 700bp for gDNA.

*Amplification of gDNA:* Genomic DNA was purified from parasites using QIAamp DNA blood mini kit (Qiagen) and used as a control.

*Quantification of Semi-quantative PCR:* Pixel intensities of scanned images were determined using ImageJ software (NIH).

A

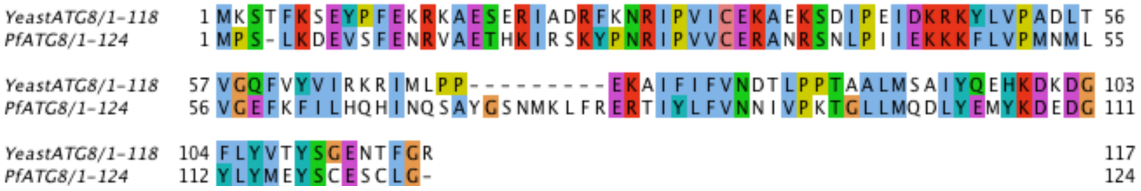


B

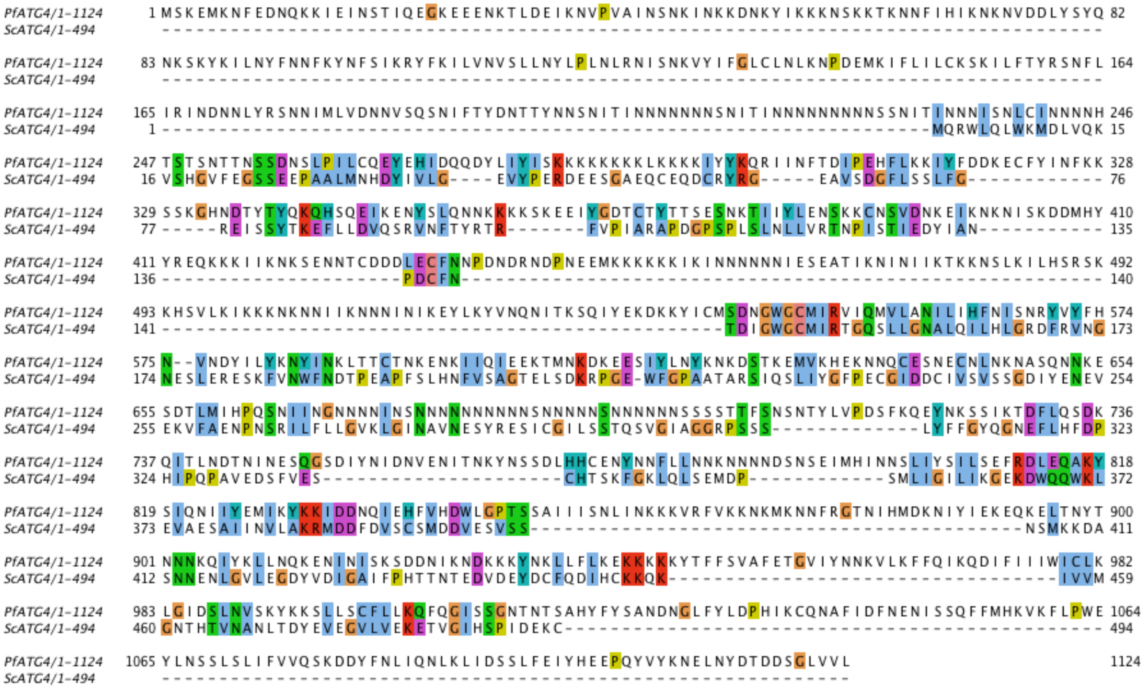

C

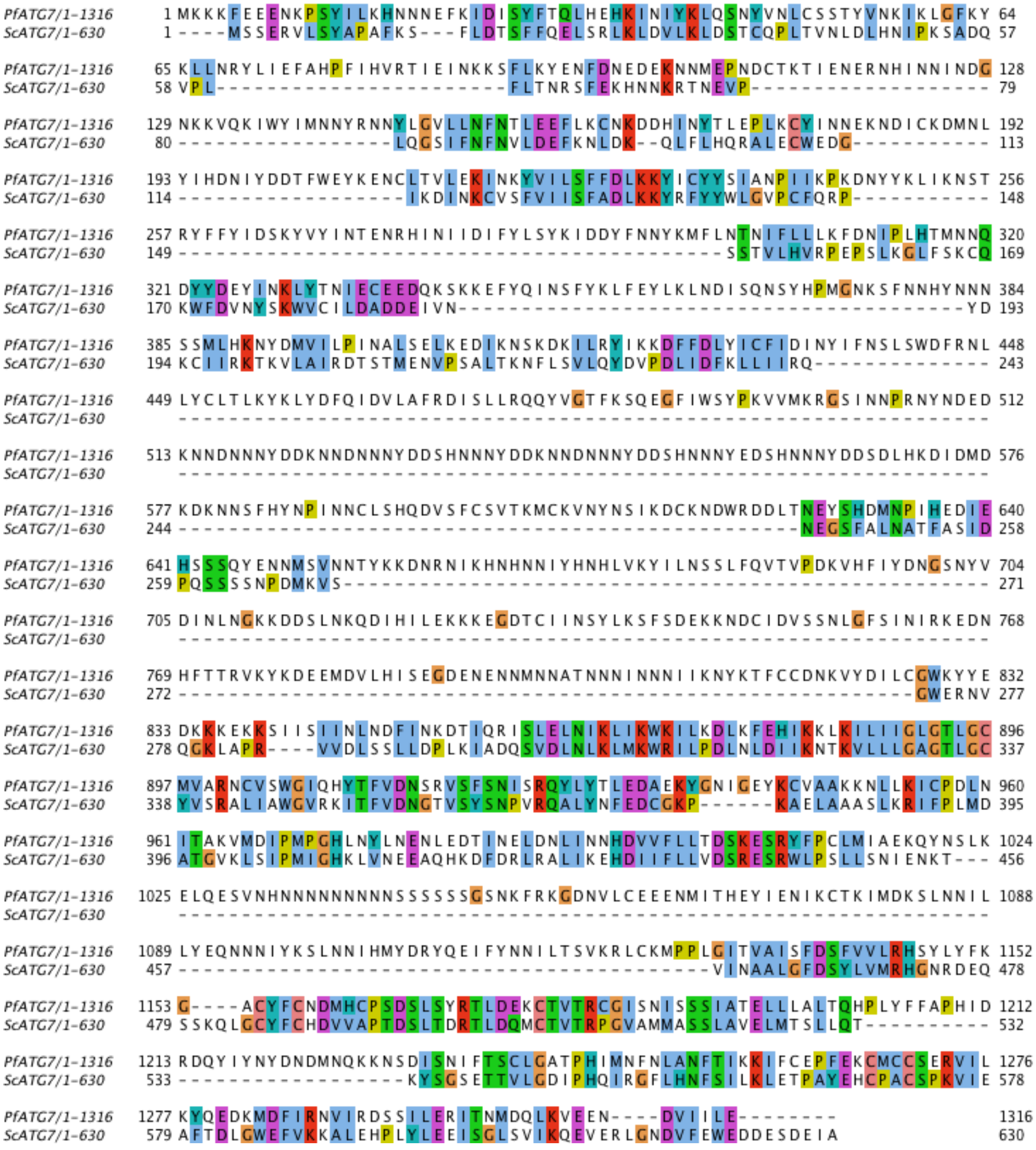

D

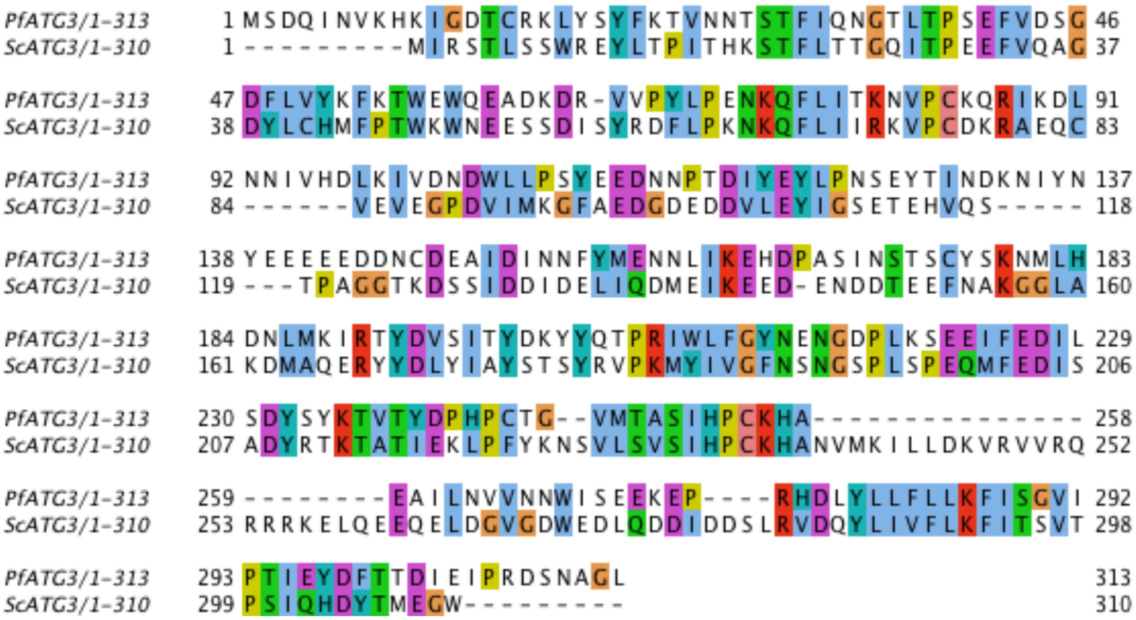


**Figure S1. Alignments show similarity between yeast and *P. falciparum* Atg proteins.** Atg8 (**A**), Atg4 (**B**), Atg7 (**C**) and Atg3 (**D**) alignments between *P. falciparum* and *S. cerevisiae* performed through ClustalW. PfAtg protein sequences identified by a PlasmoDB blastp using ScAtg protein sequences as described in M&M in the main manuscript.

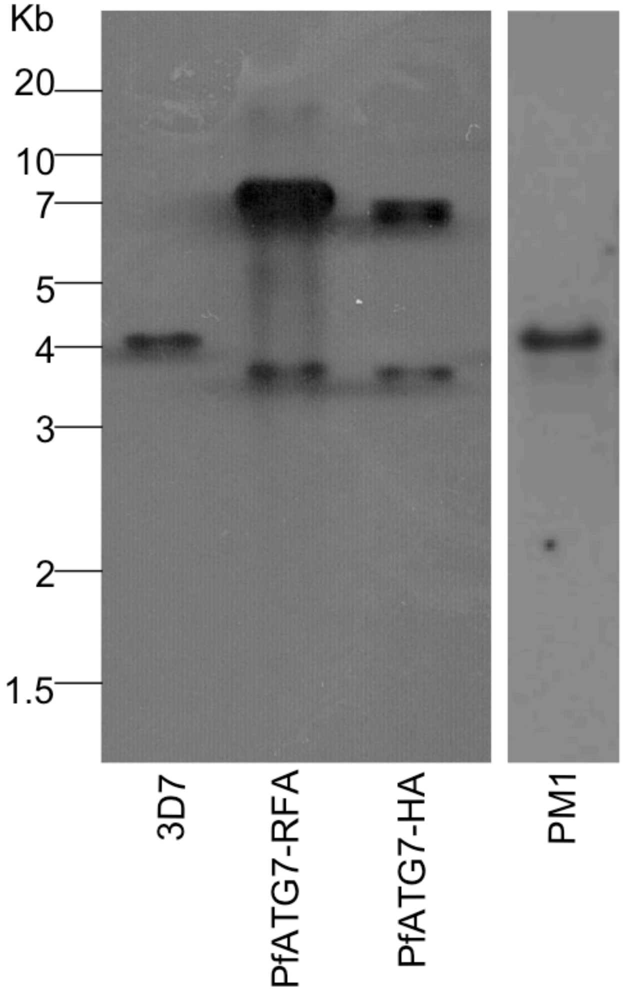


**Figure S2. Southern blot confirms integration into correct locus.** DNA was purified from transgenic parasites: PfATG7-RFA (clone A1) and PfATG7-HA (clone C1). Southern blot analyzed 1.5ug of DNA and single cross-over integrations were screened by AvrII/BclI digestion and probed using PfAtg7 ORF 3' end (1kb). The expected fragment for 3D7 and PM1 parasites is 4039 bp and the expected size fragments for successful integration of PfATG7-HA are 7653 bp and 3635 bp and for PfATG7-RFA parasites are 7528 bp and 3635 bp. Lack of wildtype locus in PfAtg7-HA and PfAtg-RFA clones indicates successful integration. High intensity band in the PfAtg7-RFA clone possibly represents concatamerization of the plasmid prior to integration into the genome.

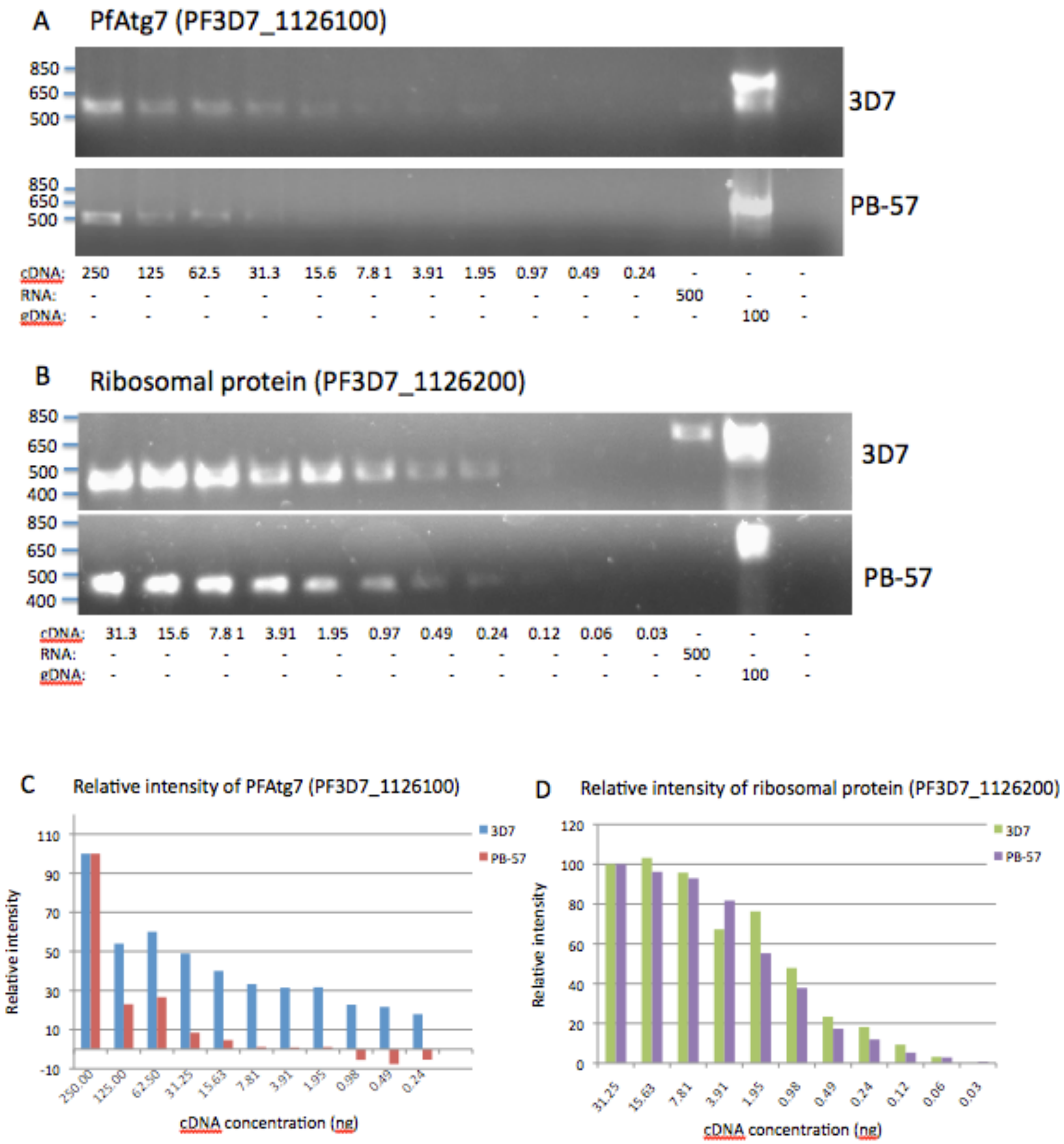


**Figure S3. Semi-quantitative PCR confirms lower expression levels of PfAtg7 in PB-57 parasites**. Standard PCR conditions were used to establish expression levels of PfAtg7 (A) and the ribosomal protein upstream of PfAtg7 (B) across a dilution series of cDNA (nanogram amounts) for 3D7 and PB-57 parasites, with quantification also shown (C,D). The ribosomal protein is used as a control as the transposable element was inserted between it and PfAtg7. Predicted sizes of amplified products: PfATG7: 566bp for cDNA, 745bp for gDNA; ribosomal protein: 464bp for cDNA and 700bp for gDNA.

| | Atg protein | *P. falciparum* orthologue | BLAST P(N) | function |
|---|---|---|---|---|
| Autophagosome formation | Atg3 | PF3D7_0905700.2 (prev PFI0280C) | 3.30E-40 | E2 type ligase for Atg8 lipidation |
| | Atg4 | PF3D7_1417300 (prev PF14_0171) | 5.00E-09 | cysteine protease for Atg8 processing and delipidation |
| | Atg7 | PF3D7_1126100 (prev PF11_0271) | 4.00E-51 | E1 type ligase or activating enzyme of Atg8 and Atg12 |
| | Atg8 | PF3D7_1019900 (prev PF10_0193) | 3.10E-24 | ubiquitin-like modifier that regulates autophagosome size |
| | | | | |
| | Atg5 | nd | | forms complex with Atg12 |
| | Atg10 | nd | | E2-like ligase Atg12-modifier protein |
| | Atg12 | PF3D7_1470000 (prev PF14_0667a) | 3.50E-09 | forms complex with Atg5 |
| | Atg16 | nd | | stabilises the Atg5-Atg12 complex |
| | | | | |
| Pre-autophagosomal structure | Atg9 | nd | | Protein of the pre-autophagosomal structure (PAS), recruites Atg8 |
| | Atg23 | PF3D7_1126700 (prev PF11_0277) | 1.20E-07 | recycles Atg9, cytoplasm to vacuole transport (cvt) protein |
| | | | | |
| | Atg1 | PF3D7_1450000 (prev PF14_0476) | 2.40E-22 | serine/threonine kinase, interacts with Atg13 |
| | Atg11 | PF3D7_0216700.2 | 4.00E-14 | interacts with Atg1, Cvt |
| | Atg17 | PF3D7_1120000 (prev PF11_0207) | 1.80E-11 | interacts with Atg1, 20 and 24 |
| | Atg31 | nd | | interacts with Atg17 |
| | Atg13 | nd | | phosphoprotein, dephosphorylated under starvation |
| | Atg2 | nd | | interacts with Atg9 |
| | | | | |
| | Atg29 | nd | | formation of PAS |
| | Atg19 | nd | | cargo receptor, Cvt |
| | | | | |
| | | | | |
| | Atg18 | PF3D7_1012900 (prev PF10_0126) | 5.20E-31 | Needed for Atg2 localisation and Atg8 recycling |
| | Atg27 | nd | | PI3P-binding protein, Cvt |
| | Atg21 | PF3D7_1012900 (prev PF10_0126) | 6.10E-12 | required to recruit Atg8 to the PAS, Cvt |

| | | | | |
|---|---|---|---|---|
| | Atg20 | PF3D7_0216700.2 | 1.40E-06 | interacts with Atg24, 17, Cvt |
| | Atg24 | PF3D7_0216700.2 | 5.80E-07 | interacts with Atg1, 17, 20, Cvt |
| | | | | |
| Upstream signalling | Bif-1 | ? | | |
| | UVRAG | ? | | |
| | Ambra1 | ? | | |
| | Bcl-2 | ? | | |
| | Vps34 | PF3D7_0515300 (prev PFE0765w) | 9.2E-75 | PI3kinase catalytic subunit, positive regulator of autophagy |
| | Vps15 | PF3D7_0823000 (prev MAL8P1.42) | 2.20E-14 | Protein kinase required for Vps34 |
| | Atg6 | nd | | Beclin 1, Interacts with Atg14, VPS34, VPS15 |
| | TOR kinase | ? | | protein kinase, negative regulator of autophagy |

**Table S1. Results of bioinformatic analysis of ATG genes in *P. falciparum.*** See M&M in main manuscript for search parameters.

1. Liu J, Gluzman IY, Drew ME, Goldberg DE (2005) The role of Plasmodium falciparum food vacuole plasmepsins. J Biol Chem 280: 1432–1437. doi:10.1074/jbc.M409740200.
